# Supplementary material for: Characterising Exciton Generation in Bulk-Heterojunction Organic Solar Cells
Source: Nanomaterials (Basel). 2021 Jan 15;11(1):209. doi: 10.3390/nano11010209 (PMC7829893; doi:10.3390/nano11010209)
Supplement: Supplementary file 1 [file nanomaterials-11-00209-s001.pdf]

Characterising Exciton Generation in Bulk-heterojunction Organic Solar Cells

Kiran Sreedhar Ram[1], Hooman Mehdizadeh-Rad[1, 2], David Ompong[1, 2], Daniel Dodzi Yao Setsoafia[1] and Jai Singh[1, 2]

[1]College of Engineering, IT and Environment, Purple 12, Charles Darwin University, Darwin, NT 0909, Australia

[2]Energy and Resources Institute, Charles Darwin University, 0909 Darwin, NT, Australia

Supplementary Materials

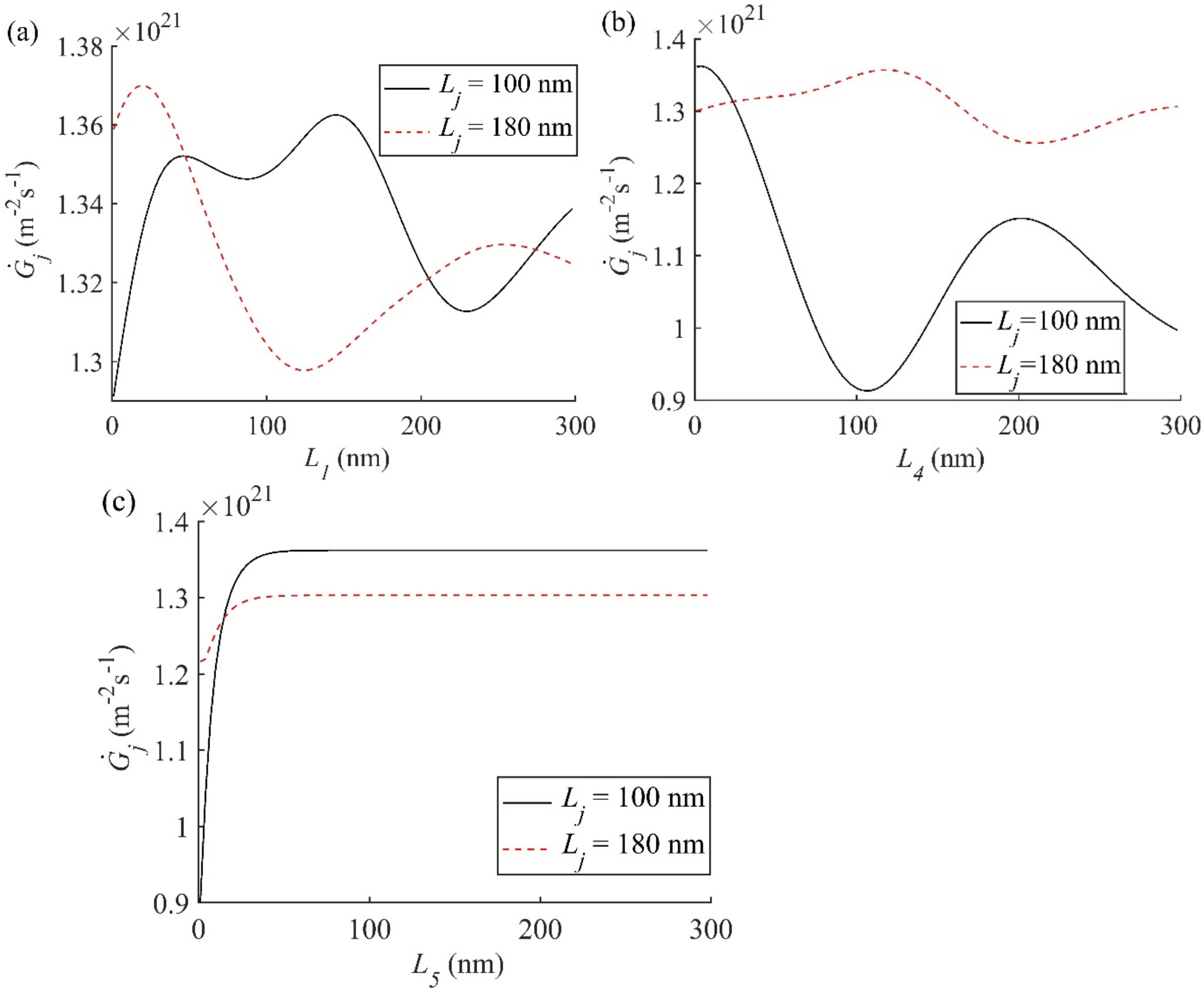

Figure S1: For OSC2, $\dot{G}_j$ is plotted for two active layer thicknesses L_j = 100 nm and 180 nm *as function of the thickness of (a) L_1 of layer 1 (ITO), (b) L_4 of layer 4 (PFN-Br), (c) L_5 of layer 5 (Al).*

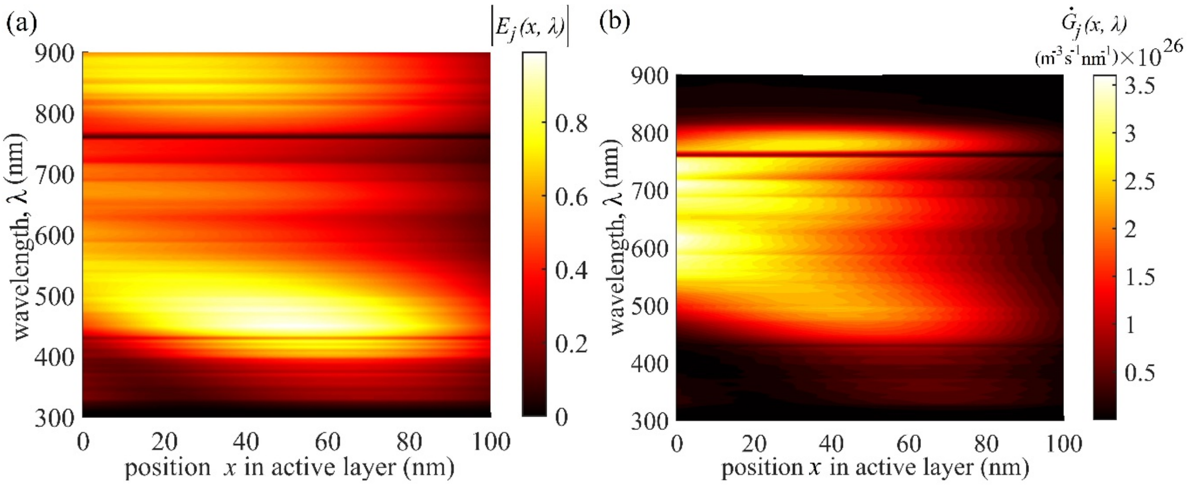

Figure S2: (a) The contour plot of $|E_j(x, \lambda)|$ from Eq. (5) in the active layer of OSC2 for L_j=100 nm. The brighter spots represent regions of the constructive interference of electric field of EM waves within the active layer and the darker spots represent regions of destructive interference. (b) The corresponding $\dot{G}_j(x, \lambda)$ contour plots from Eq. (3), showing the brighter spots as positions of higher $\dot{G}_j(x, \lambda)$ and darker spots that of lower $\dot{G}_j(x, \lambda)$.

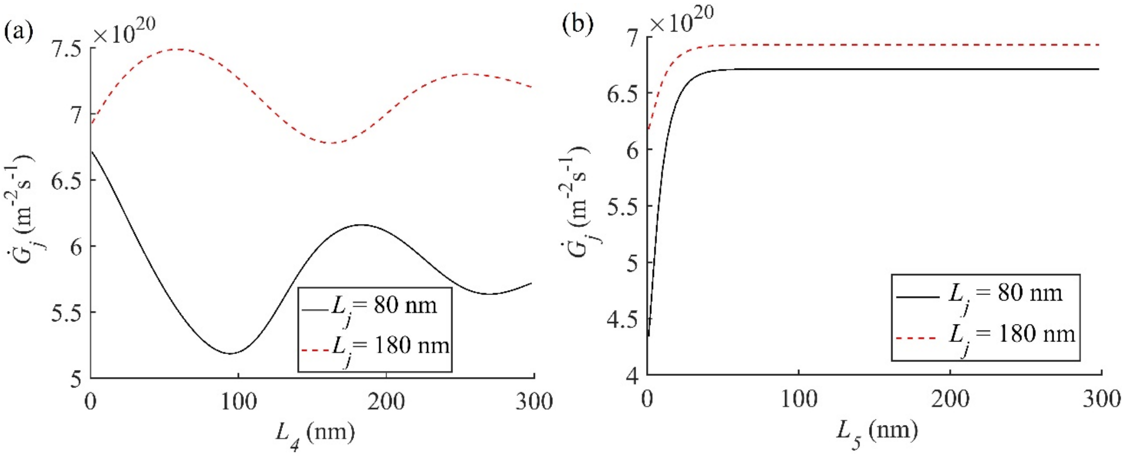

Figure S3: For OSC3, $\dot{G}_j$ is plotted for two active layer thicknesses L_j = 80 nm and 180 nm, *as function of the thickness of (a) L_4 of layer 4 (LiF), (b) L_5 of layer 5 (Al).*

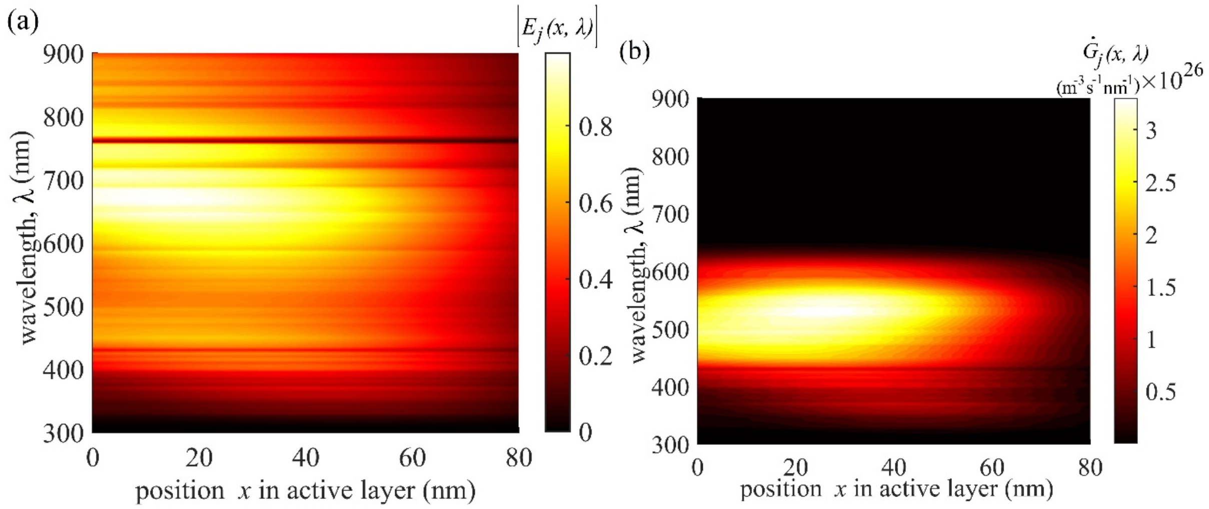

Figure S4: (a) The contour plot of $|E_j(x,\lambda)|$ *from Eq. (5) in the active layer of OSC3 for* L_j*=80 nm. The brighter spots represent regions of the constructive interference of electric field of EM waves within the active layer and the darker spots represent regions of destructive interference. (b) The corresponding* $\dot{G}_j(x,\lambda)$ *contour plots from Eq. (3), showing the brighter spots as positions of higher* $\dot{G}_j(x,\lambda)$ *and darker spots that of lower* $\dot{G}_j(x,\lambda)$*.*
